# Does Amyotrophic Lateral Sclerosis (ALS) Have Metabolic Causes from Human Evolution?

**DOI:** 10.3390/cells14211734

**Published:** 2025-11-05

**Authors:** Michael Spedding

**Affiliations:** Spedding Research Solutions SAS, 78110 Le Vesinet, France; michael@speddingresearchsolutions.fr; Tel.: +33-637059188

**Keywords:** evolution, ALS, lactate, CMAH, exercise, glycosphingolipids, ageing, sialome, GM1

## Abstract

As so many drugs have failed in ALS a new approach is needed. The author proposes that recent human genetic variants may play major roles in the disease, changing metabolism. Evolution of hominins was accelerated 3–2.5 Mya, by cytidine monophospho-N-acetylneuraminic acid hydroxylase (CMAH) becoming a unitary pseudogene after a pathogenic infection, changing the sialome, and hence metabolism, brain development and neuromuscular junctions (NMJs). This was when hominins evolved to run in Africa and develop bigger brains. Deletion of CMAH in mice allows them to run for longer (~50%). The enzyme CMAH is critical for the sialome, particularly the neurotrophin GM1, a critical hub for viral infection and for NMJ stability, but which is lost from NMJs at the beginning of denervation, probably due a 10-fold increase in spinal cord glucosylceramidases (non-lysosomal GBA2). A GBA2 inhibitor, ambroxol, is currently in phase II for ALS. Human-specific GM1 may be critical for human evolution, lactate metabolism and ALS. Lipid/lactate metabolism changed to support these evolutionary changes and lactate is a major body/brain fuel, but compromised in ALS patients and a marker of disease progression. Recent progress in sports science involving lactate metabolism and human performance may also be relevant to ALS therapies, and incidence.

## 1. Introduction

Amyotrophic lateral sclerosis (ALS) is a disease affecting upper and lower motor neurons, causing progressive denervation associated with loss of motor neurons, leading to death from respiratory failure: the incidence is ~10/10,000 in the USA with survival usually between 1 and 5 years [1]. Denervation may start in limbs, (limb-onset, two thirds of patients) or with difficulties in speaking or swallowing (bulbar-onset). Most cases have no apparent genetic cause (sporadic ALS, ~80–90%), but many genetic variants have been described with the most common genes affected being *C9orf72*, *SOD1*, *TARDBP* and *FUS*: variants of these genes are associated with an earlier onset of symptoms. However, a common factor in 97% of cases is cytoplasmic aggregation of the nuclear RNA- and DNA binding protein, TAR DNA-binding protein-43 (TDP-43), which has attracted massive attention, although mis-localization may occur in non-nervous tissue well before symptoms of ALS [2].

ALS has very few therapeutic options in terms of drugs other than riluzole which may prolong survival by ~10%, although the antisense oligonucleotides Tofersen and ION363/Jacifusen have been able to reverse the phenotype in a few patients with aggressive mutations of Cu/Zn superoxide-dismutase1 (*SOD1)* and fused in sarcoma RNA-binding protein (*FUS*) [3,4,5]. More than 76 drugs have been used in clinical trials until 2021, without success [6], and while more than 25 are now in clinical development, nearly all are targeting specific but non-overlapping mechanisms, showing that there is no overall consensus on ways forward. This article presents a different perspective, whereby a pathological infection, perhaps viral, ~3 Mya, selected humans with a pseudogene (CMAH) which modified a critical neurotrophin (GM1, important for metabolism) enchaining events leading to the evolution of modern humans and their capacity to run, but potentially increasing the risk of motor neurone diseases.

While clinical diagnosis of ALS has progressed successively with El Escorial [7], Gold Coast [8] and Miami [9] recommendations, the molecular drivers of sporadic ALS remain obscure. In nearly all cases of ALS, the protein TDP-43 is mislocalised from the nucleus to the cytoplasm forming aggregates, disrupting RNA metabolism, and splicing, but aggregates appear in non-CNS tissue years before diagnosis [2], and the links with ageing are not clear. Mutations of *TARDP, C9orf72* and *SOD1* genes are very penetrant in ALS with variants taking between two and four of the six steps in disease progression, one step being ageing [10,11]. While these variants are commonly introduced into animal models, SOD1 mouse models have been criticized, as positive results in the models were not confirmed in the clinic. However, when ALS-TDI tested thousands of SOD1^G93A^ mice with a rigorous protocol in a drug screening campaign, no drug worked, indicating the problem was with the drugs rather than the model [12]. Many animal models based on ALS variants have been made, but with very varied disease penetrance, and only a few have a robust phenotype of denervation. Weak disease phenotypes may mislead target priorities for drug discovery, if purely biochemical pathways are targeted which are not directly related to phenotype and the critical paths to ALS progression.

So, which molecular mechanisms are sufficiently powerful to initiate or spread ALS, downstream from the major mutations? The major mutations have been well defined [13], but these do not account for sporadic ALS, or where multiple mutations and/or environmental factors converge on key pathological mechanisms. Is ALS specific to humans? While multiple animal models exist, usually based on introducing these highly penetrating mutations, sporadic ALS is very rare in the animal kingdom [14] except for degenerative myelopathy in dogs, yielding ALS-like symptoms, but which are also associated with *SOD1* mutations (e.g. *SOD1A^E40K^*, [15]), but these mutations can be bred out, unlike human sporadic ALS. Are there human-specific mechanisms, present in all humans, which drove human evolution, which may allow drug targeting?

## 2. Evolution and Glycosphingolipids, Viruses and Metabolism

The evolution between pathogens and organisms is continuous, but certain major systems have proven critical in the past for human evolution. Glycans decorate the cell surface, frequently tipped with sialic acids, which are monosaccharides with a nine-carbon backbone, forming the sialome. The sialome has been likened to the canopy of a forest, with immense diversity and 10–100 million sialic acids/cell [16]. For example, a sialic acid linked to two monosaccharides may lead to >113,000 possible linear sialyltrisaccharides [17,18] and a pentasaccharide of D-hexoses has 2.6 × 10^9^ possible structures [19], rendering molecular definition difficult, although nature has evolved highly efficient recognition systems. Sialic acids bind Siglecs (sialic acid-binding immunoglobulin-type lectins) located on immune cells, modulating immune responses.

**Figure 1:**
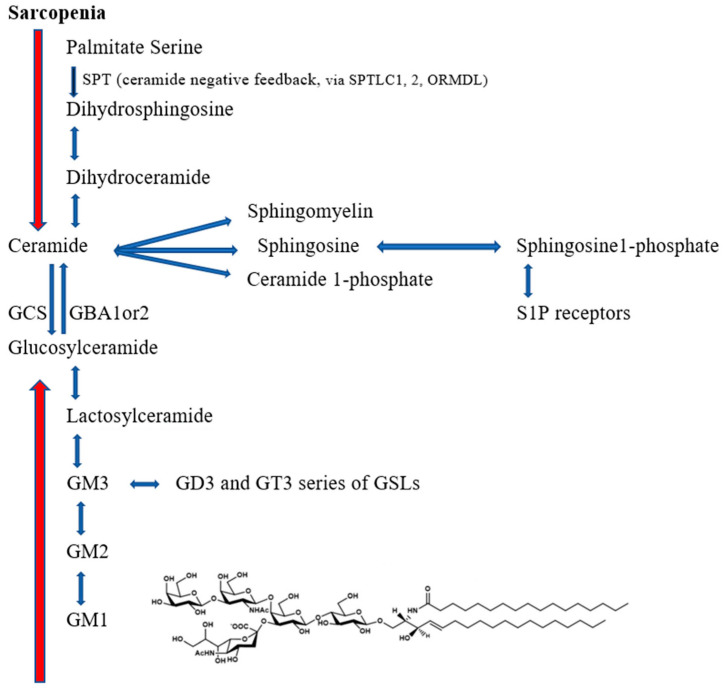
Simplified scheme of glycosphingolipid synthesis and degradation (for more complete schemes with all the GSLs/enzymes, see [20,21,22]). Synthesis is in the endoplasmic reticulum then Golgi apparatus and degradation is predominantly in lysosomes (with specific mutations in degradative enzymes leading to lysosomal diseases. Ceramide exerts negative feedback on sphingolipid synthesis at serine *C*-palmitoyltransferase (SPT) via its two heterodimeric subunits encoded by *SPTLC1* or *SPTLC2* genes or regulatory ORMDL proteins. Glucosyl ceramide synthase (GCS) and glucosylceramidases (lysosomal, GBA1 and non-lysosomal, GBA2, form critical nodes for both viral infection, and neurodegeneration (see text). The formation of galactosylceramide by UDP-galactose ceramide galactosyltransferase may also occur in the endoplasmic reticulum/Golgi. The structure of the human form of GM1 is shown, as an example of a Neu5Ac ganglioside.

An example of a sialic acid-tipped glycosphingolipid (GSL) is GM1 (Figure 1). GM1, GD1a (a reserve pool for GM1), GD1b, and GT1b comprise most of adult human brain gangliosides (gangliosides are GSLs tipped with a sialic acid) [20]. The GSLs and gangliosides are critically controlled during the stages of development [20], because of their recognition capacity as the oligosaccharide of the ganglioside GM1 has ~16 potential hydrogen bonds to interact with adjacent proteins (Figure 1).

The synthesis and degradation of GSLs and gangliosides is complicated, so only a simplified scheme of glycosphingolipid synthesis is shown in Figure 1 (for a more complete scheme with all the GSLs/enzymes, see [20,21,22]). Synthesis is in the endoplasmic reticulum/Golgi apparatus and degradation predominantly in lysosomes (with specific mutations leading to lysosomal diseases, e.g., GBA1: Parkinson’s disease, Gaucher’s disease). The different pathways for synthesis and degradation are critical to understanding the GSL/ganglioside metabolism, as degradation is mainly via lysosomal metabolism and mutations in the enzymes causing degradation may cause very high levels of upstream GSL/gangliosides with their distinct toxicity profiles, which may mask changes in synthesis or non-lysosomal degradation. For example, GM1 gangliosidosis, caused by mutations in *GLB1* leading to deficiencies in lysosomal-β-galactosidase, massively accumulates lysosomal GM1. Thus, on the one hand nizubaglustat, a glucosylceramidase synthesis inhibitor, is in clinical trials for GM1 gangliosidosis, whereas liposomal GM1 is in clinical trials for Parkinson’s disease.

**Figure 2:**
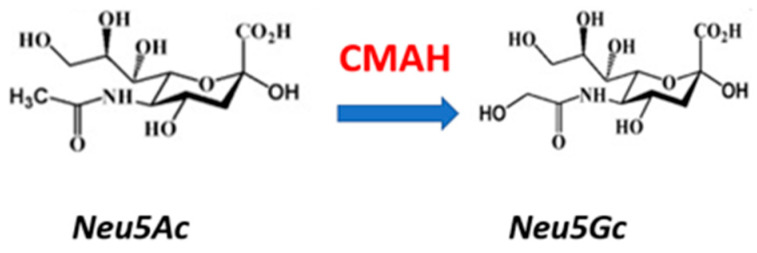
Modification of the sialic acid Neu5Ac to Neu5Gc by cytidine monophospho-N-acetylneuraminic acid hydroxylase (CMAH).

However, the sialome for humans is distinct from most non-human primates in one major aspect, which may have been critical for human evolution. The enzyme, cytidine monophospho-N-acetylneuraminic acid hydroxylase (CMAH) became a pseudogene, approximately 3.2–2.5 Mya, modifying the sialome, as described by Varki [16,18,23,24,25]. This enzyme hydroxylates N-acetylneuraminic acid (Neu5Ac), yielding N-glycolylneuraminic acid (Neu5Gc), which is a terminal sialic acid in most mammals and in old world non-human primates, such as chimpanzees [26]. Since CMAH became a unitary pseudogene in human [25], by losing the N-terminal 104 amino acids, via the deletion of one exon [25,27], there is little detectable Neu5Gc in humans: thus Neu5Ac compensates for Neu5Gc loss. However, Neu5AC by losing an oxygen, loses a potential hydrogen bond compared with Neu5Gc and is more hydrophobic [28] (Figure 2).

Humans have some Neu5Gc from dietary input from meat and dairy produce, resulting in varying degrees of antibodies to Neu5Gc. A high intake of red meat, has been linked with a higher risk of developing dementia and poorer cognition in a large study of US adults [29]. CMAH is downregulated in the brains of most mammalian species (<3% Neu5Gc) and genetic re-expression specifically in the brain of CMAH null mice results in neurodegeneration and abnormal axon myelination [30]. It has been postulated that complete inactivation in hominins of CMAH led to subsequent human brain expansion [31]. The specificity of probes exploring these changes has been defined [32]. These changes in hominins predate divergence from Neanderthals and Denisovans by ~0.6 Ma, and may have taken place via complicated immune interactions with pathogens, as pathogens may have first eliminated hominins with Neu5Gc, and subsequently mutated to interact with Neu5Ac rather than Neu5Gc over the last 2.5 million years [33,34]. As Neanderthal introgression of DNA in the human genome does not contribute to neurodegenerative disease [35], this further argues for CMAH being a potential ‘upstream’ risk factor for human-specific diseases.

The change of Neu5Gc to Neu5Ac in gangliosides has also resulted in corresponding changes in the ganglioside receptors, Siglecs, which are important for the immune system and some ALS-related targets such as Siglec-4 [34]. Siglec-4 (myelin-associated glycoprotein) is not linked to immune cells, but rather to oligodendrocytes and Schwann cells, controlling the tight links between axons and myelin (myelin-axon spacing), which are disrupted in multiple diseases, including ALS. Following myelin disruption, Siglec-4 inhibits axonal outgrowth (https://glycopedia.eu/echapter/article-introduction-3/article-mag-siglec-4/ accessed on 1 October 2025). Siglec-4 is highly expressed in myelin, inhibits axon growth, by binding with the gangliosides GD1a and GT1b. GD1a, by having an additional sialic acid, which is removed by NEU3 neuraminidase, is a reserve pool for GM1 [36,37]. Co-crystallization of Siglec-4 and NeuAc-N-acetylactosylamine has shown how the arginine118 of Siglec-4 interacts with the carboxylic acid of Neu5Ac and allowed the definition of the intricate hydrogen bond network, with Siglec-4 forming a dimer to control outgrowth inhibition [38].

Gangliosides and GSLs, with cholesterol, are critical components of lipid rafts, and their kinetics in rafts have been studied [39]. Human enveloped viruses, including influenza and SARS-CoV-2, now interact with Neu5Ac (with less affinity for Neu5Gc), in the mucus (decoy receptors), or on lipid rafts. GM1, GD1a, and GM3 are co-receptors for SARS-CoV-2, with ACE-2 [40,41,42]. Viruses with affinity for Neu5Gc have been proposed to play a role in human evolution ~3 Mya, presumably by allowing the selection of hominins with the CMAH pseudogene [43]. Apart from the initial stages of cell entry, glucosylceramide synthase and glucosylceramidases play multiple roles in enveloped virus replication [44,45,46,47,48] and are therefore critical hubs for both viral infection and neurodegenerative diseases (Figure 1 and Figure 2), controlling the synthesis and breakdown of GSLs and their interaction with lipid rafts, which have been directly linked to neurodegeneration in ALS [49]. Infection with a β-coronavirus in mice leads to the degeneration of motor neurons and fragmentation of NMJs [50], but while a slight increase in the incidence of ALS post-COVID has been reported [51] this may be due to poorer healthcare during the pandemic. There is no clear clinical link yet between viral infection and ALS, although long-term mitochondrial dysfunction may persist after viral infection [52], potentially accounting for a step in ALS progression, along with ageing [10].

The period of ~2.5 Mya was when hominins evolved to run, and also enlarge their brains, and was a time of climatic change in Africa [53,54], with very major changes in human physiology, metabolism and subsequent susceptibility to diseases [55]. The development of speech would have involved the specialization of bulbar motor neurons. Some of these evolutionary changes are shown in Table 1. In reverse, CMAH deletion in mice has major peripheral metabolic effects, with a higher maximal respiratory capacity and an improved skeletal muscle capacity for oxygen use which lead to a highly significant increases in time running on a forced treadmill for untrained animals and in time voluntarily wheel running [56], consistent with the role in human evolution. However, deletion of CMAH may worsen the severity of disease progression in mdx mice, and also in a-sarcoglycan-deficient mice, raising the possibility of involvement in muscular dystrophy [57] and in other diseases.

## 3. Glycosphingolipids and Gangliosides in ALS

**Figure 3:**
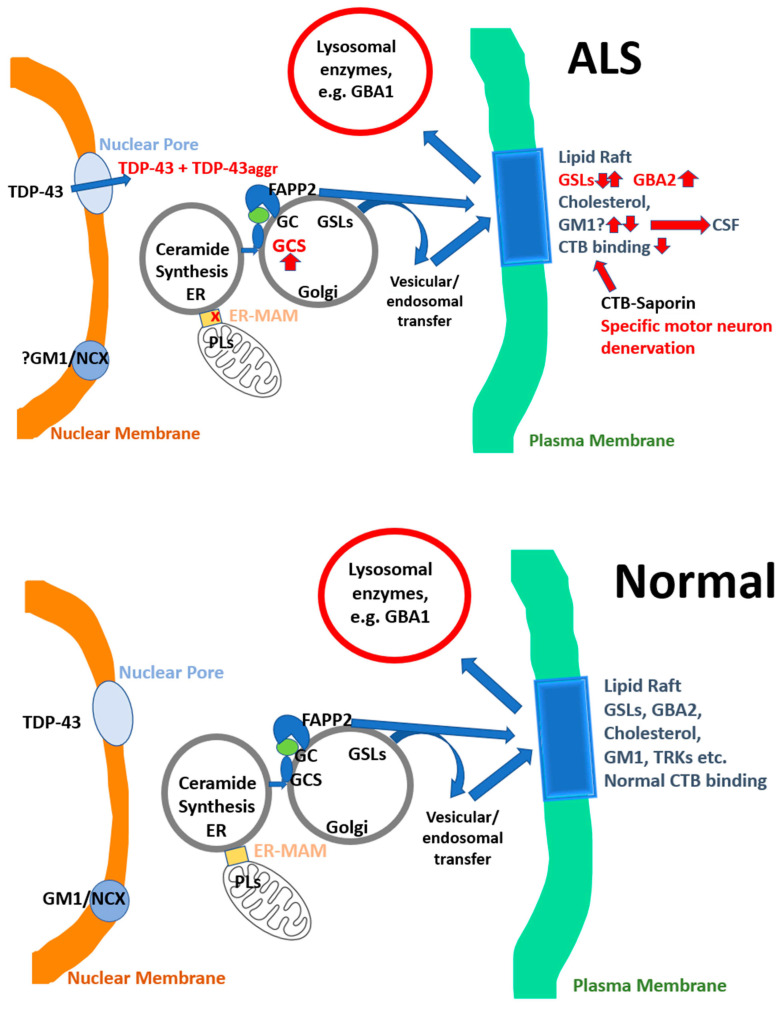
Schematic changes of glycosphingolipid and ganglioside metabolism in motor neurons comparing normals with ALS (see text). CTB: cholera toxin β-subunit, CTB-S: cholera toxin β-subunit bound to saporin, ER-MAM, endoplasmic reticulum mitochondria-associated membrane, FAPP2: phosphatidylinositol-four-phosphate adapter protein 2, GalCer: galactosyl ceramide; GC: glucosyl ceramide, GBA1: lysosomal b-glucosylceramidase, GBA2: non-lysosomal β-glucosylceramidase, GCS (or UGCG): Ceramide glucosyltransferase, GM1: monosialotetrahexosylganglioside, GSL: glycosphingolipid, NCX; nuclear Na^+^/Ca^2+^ transporter, TDP-43: transactive response DNA binding protein of 43 kDa, PL: phospholipids.

While serine palmitoyl transferase (SPT) is rate-limiting for sphingolipid synthesis (Figure 1), the triad of GCS, GBA1, GBA2 and are responsible for the synthesis and breakdown of GSLs with different cellular localisations, and roles (Figure 1 and Figure 3). Enzyme activity of both glucosylceramide synthase (GCS, encoded by the *UGCG* gene) and of non-lysosomal glucosylceramidases (GBA2) in spinal cord and muscle are increased from the beginning of denervation in multiple animal models of ALS [63,64,65,66,67,68,69]. The *UGCG* gene (ceramide to glucosylceramide) and its immediate downstream gene *B4Galt5* (glucosylceramide to lactosyl ceramide) have recently been shown to be ‘super-enhancers’ changing the cell phenotype, being essential for NK cells and CD8^+^ cytotoxic cells in their response to viral infection [70,71]. This effect is glucose-dependent, leading to increased synthesis of GSLs which are transferred to lipid rafts, increasing T cell signalling and cytotoxic function [72]. Furthermore, the activity of GBA2 is increased 8–10 fold in spinal cord at the beginning of denervation in SOD1^G86R^ mice [63] (Figure 3) and GCS has also been reported to be elevated in this model [65]: the question of which change may be compensatory has been only partly resolved, and intracellular GSL location may be a critical factor (Figure 3), potentially leading to different compensatory mechanism in disease states. Galactosylceramidase, GBA1 and GBA2 activities were increased up to 3-fold in the spinal cord of ALS patients, when ceramide and GSLs were increased, albeit ceramides appear to be preferentially increased in muscle [65,67]. In SOD1^G93A^ mice, inhibition of GCS accelerated disease progression, whereas infusion of GM3 slowed progression [67]. Inhibition of GCS facilitated progression of denervation in ALS models, and also delayed recovery after sciatic nerve crush [65,67], implying maintaining GSL and ganglioside levels is important for maintaining innervation. There remains some controversy in the literature as to whether glucosylceramide, the key building block for many GSLs and gangliosides, is elevated [64,73] or reduced [65] in spinal cord of models of ALS [65,67]. However, a difficulty in the litterature is that glucosylceramides are not readily separated from galactosylceramides in standard metabolomic studies (unless techniques such as HILIC–ESI–MS/MS are used [74]). Galactosylceramides are highly enriched in spinal cord with sulfatide (3-O-sulfogalactosylceramide) which may form glycosynapses between oligodendrocytes and myelin [75]. Furthermore, both enzymes, GBA1 and GBA2 have both transglycosylation and transgalactosylation activity [74] so they not only degrade glucosylceramide and galactosyceramide but also transfer glucose or galactose to specific acceptors, such as cholesterol, which would have major impact on lipid raft stability. The inhibition of GCS also increases ceramide levels [67], as does the marked increase of GBA2 activity in the spinal cord, allowing the hypothesis that GBA2 inhibitors will be beneficial in ALS (see below). In this respect GBA2 has been linked to movement disorders and defects in motor neuron function [76,77,78].

## 4. Cholera-Toxin Binding as a Tool to Study GM1, and Fucosylated Structures, and to Cause Denervation

Neu5Ac and GM1 are also the main targets for cholera toxin β-subunit (CTB), developing Vibrio cholerae virulence, specific to man [79,80]. The binding of GM1 to CTB is one of the highest affinity carbohydrate-protein interactions known, predominantly to the terminal sialic acid (Neu5Ac) and adjacent galactose residues [81] and has been used to assess GM1 levels.The evolutionary adaptation of V. Cholerae to man, and to Neu5Ac, and its virulence, has been described in detail: CTB binds to GM1 in lipid rafts and is internalized with GM1 [82]. CTB can be used to label GM1, although precautions must be taken [83], and fucososylated/galactosylated substances can also bind CTB with relatively high affinity [84,85,86]. However, fucosyl-GM1 has very low expression in normal tissue, albeit high in cancers, particularly small cell lung cancer [87,88]. Nevertheless, protein-O-fucosylation is common.

The intramuscular injection of cholera toxin bound to saporin (CTB-S) is now a well-established method for inducing specific motor neuron death by retrograde suicide transport to nerves expressing GM1 [89] and has been widely used as a model for motor neuron diseases, by localizing motor neuron dysfunction and death to specific neuron tracts, thereby eliminating systemic effects [90,91,92]. CTB-S causes respiratory failure via phrenic motor neuron death in rats following intrapleural injection [93]. Exercise (access to running wheels) has been shown to be partially protective by increasing dendritic arbors of motor neurons in rats injected into the vastus medialis with CTB-S [94]. Intralingual CTB-S in rats reduces hypoglossal motor neuron survival, swallowing and changes tongue morphology in a way similar to bulbar ALS [92,95]. Again, tongue exercise, with high-repetition/low resistance exercise was beneficial in this model [96]. Thus, CTB may be used as a surrogate marker of GM1, but CTB-S also causes specific motor neuron death, presumably by binding to, and downregulating, GM1, and possibly other fucosylated targets: powerful evidence linking loss of GM1 to denervation in ALS.

As viruses bind to GM1, and related GSLs, in lipid rafts to gain entry into cells, antibodies to GM1 may be formed which cause multifocal motor neuropathy [97] by affecting motor neurons [98] and are also involved in cases of post-viral Guillain-Barré syndrome, and peripheral neuropathies [99], also affecting upper motor neurons [100]. In a study of 103 patients, serum IGM anti-GM1 antibodies were found in 46% of patients with lower MND, 80% of patients with motor neuropathy, and 18% with classical ALS [101]. While other studies have reported similar findings [102,103,104,105,106,107], more recent studies have shown that ALS patients with elevated ganglioside antibodies had the same progression as patients without antibodies, and an overall conclusion was that sporadic ALS patients hardly differed from controls, whereas in multifocal motor neuropathy, treatable by immunosupression, antibody detection was useful [105,108]. Nevertheless, these studies reinforce the link between motor neurons and GM1, and GD1a, a reservoir for GM1.

GM1 declines in the human brain with age [109], and also in mouse brain and periphery [110]. GM1 injection was much used in the treatment of peripheral neuropathies 30 years ago, but fears of Guillain-Barré syndrome (probably linked to impurities in synthesis) lead to its withdrawal [111]. Widespread deficiency of GM1, and GD1a, has been claimed to be a cause of Parkinson’s disease, linked to aggregated α-synuclein (reviewed in [112,113,114]). GM1 binds to α-synuclein, resisting aggregation [115]. The multiple GBA1 variants linked to Parkinson’s disease, which are the greatest genetic cause of the disease, exacerbate this aggregation [116,117,118]. GM1 infusion has been investigated in Parkinson’s disease [119], but hindered by poor passage of the blood-brain barrier. More lipophilic analogues of the oligosaccharide portion of GM1 are being investigated [120]. As GM1 is predominantly broken down in lysosomes by β-galactosidase, mutations in *glb1* causes massive accumulation of GM1 and GM1-gangliosidosis, associated with neurodegeneration, so the precise control of the synthesis and degradation of GM1, and related gangliosides, appears critical for their multiple functions.

## 5. Is GM1 Directly Implicated in ALS?

The main gangliosides located in unmyelinated bovine splenic nerve are GM1, GD1a, GT1b [121]. GM1 is necessary for the stability of NMJs, but lost in denervation [66,73] (Figure 3). A gene necessary for the synthesis of GM1 and other gangliosides, *B4GALNT1*, encoding β-1,4-N-acetylgalactosaminyl transferase, was reported to be a putative locus for ALS in a genome-wide association of European and Chinese populations [122]. GM1 is protective against glutamate-induced toxicity in motor neurons from wild type mice and SOD1^G93A^ mice, protecting against mitochondria loss and mitochondrial free radical production, SOD1 aggregation and TDP-43 mis-localization: these effects are dependent on the oligosaccharide part of the molecule [123]. While GM1 is a component of lipid rafts, the oligosaccharide portion can bind to a specific pocket on TrkA receptors, thereby synergizing with NGF [124]. Furthermore, a lipophilic site in the membrane-spanning portion of TrkA binds to the ceramide portion of GM1 and is required for bringing TrkA to the cell membrane, necessary for NGF binding and neurotrophic effects [125]. Binding of GM1 is specific for TrkA and TrkB and this interaction is not shared with other gangliosides [125,126]. GM1 has been proposed to be a key factor in protecting against multiple forms of neurodegeneration [127], particularly Parkinson’s disease [128]. Moreover, cholera-toxin binding can be visualized pre-synaptically in NMJs and is lost at the very start of denervation in SOD1^G86R^ mice, at 95 days [63,66,73] (Figure 3). At the same time, non-lysosomal β-glucosylceramidase (GBA2) activity is increased 8–10-fold at the start of denervation in the spinal cord of these mice, breaking down GSLs [63] (Figure 3). The lysosomal β-glucosylceramidase (GBA1), where mutations are the main genetic cause of Parkinson’s disease, was not increased in the muscle nor spinal cord of SOD1^G86R^ mice (Figure 3) [63]. The GBA2 inhibitor, and GBA1 chaperone, ambroxol, a generic mucolytic drug, was protective against denervation and loss of CTB in NMJs of SOD1^G86R^ mice and also in a model of sciatic nerve crush [63]. Ambroxol also increased NMJ formation, in a concentration-dependent manner, from spinal cord explants on myoblasts in tissue culture [63]. The cause of the increase of GBA2 activity in spinal cord at the beginning of denervation is not known but will profoundly affect levels of GSLs, including GM1 (Figure 3), and also increase intracellular ceramide levels [64,65,66,67,68,129]. Furthermore, as GBA2 can transglycosylate/transgalactosylate cholesterol, this may explain some aspects of disorders of cholesterol metabolism in ALS [74] and affect lipid raft stability [130]. GM1 and GlcCer are elevated in the CSF of ALS patients [131], perhaps as a result of cellular damage. These effects are listed in Table 2.

## 6. Links with BDNF

We [54] and others [58] had previously argued that brain-derived neurotrophic factor (BDNF) may have been a motor for human evolution 3–2.5 Mya, as BDNF is produced by exercise, activates tropomyosin receptor kinase-B (TrkB) and the neurotrophic cascade, increasing neuronal connectivity, synapse development and NMJs [54]. However, GM1 binds to TrkA and TrkB receptors, thereby having neurotrophic effects directly [126,140] and is an important driver of neuronal differentiation. There are two specific sites for GM1 on the TrkA receptor, essential for TrkA movement to the cell surface and lipid raft localization [124]. While the effects of GM1 on TrkB receptors have been less studied, the antidepressant effects of GM1 in mice are mediated via BDNF [141]. GM1, and related glycosphingolipids may therefore have been upstream to neurotrophins in human evolution. Furthermore, BDNF, but not other neurotrophins, increases brain mitochondrial respiratory coupling index (RCI) a measure of mitochondrial efficiency, which is directly related to some of the neuroprotective effects of BDNF, via Trk-B, mitogen-activated protein (MAP) kinases, B-cell lymphoma-2 (Bcl-2) and voltage-dependent anion channel 1 (VDAC1) in mitochondria [142,143]. The effects on RCI were blocked by interleukin 1β (IL1β) and nerve-growth factor (NGF), both of which extended ibotenate-induced grey and white matter lesions in mice whereas BDNF was protective [142]. Mutant SOD1 may disrupt these effects of BDNF on respiratory efficiency, as it forms a complex with Bcl2 and VDAC1, causing mitochondrial hyperpolarization and loss of motor neurons [144]. Although the neurotrophic effects of BDNF on isolated motor neurons are robust, attempts to use BDNF therapeutically in ALS have not been successful [145], although BDNF serum levels are lower in ALS patients as they progress [119].

Human cortical neurones have distinct properties compared with those from other primates, in their metabolism, complexity and action potential generation [146,147,148], although the roles of Neu5Ac-GM1 and BDNF in this differentiation have not been defined. Primary motor cortex, and its gene expression, has been studied in detail across species, with an expansion of the ratio of oligodendrocytes to axons in man [149].

The links between mitochondrial metabolism and evolution of the human brain have been clarified recently, in that the areas which have evolved most recently have the highest number of mitochondria, according to the human MitoBrainMap [150]. However, reduction of mitochondrial function is important for the extending the window of human neurogenesis in brain development, via doubling of copy number of Olduvai protein domains from *NBPF* genes (1q21.1, paired with *NOTCH2L*), compared with chimpanzee [61]. Similarly, *SRGAP2C* delays dendritic spine maturation, via a synaptic protein, CTNND2 [146,147]. The protein from the human-specific gene *ARHGAP11B,* when transported into the mitochondrial matrix, stimulates glutaminolysis, in conjunction with GLD2, and has been linked to the increase in neocortex size [62]. A hominid specific gene family *LRRC37* is duplicated and *LRRC37B* is highly expressed in the axon initiation segment, as are sodium channels, and the gene product binds to FGF13A and Na_v_1.6, reducing excitability. Lactate metabolism is increased in the developing human brain with a greater reliance on aerobic glycolysis [146]. It will be important to define how many of these changes are secondary to CMAH becoming a pseudogene, linked to the multiple other genetic changes which have been extensively reviewed by Varki’s group [151].

## 7. Ceramides and ALS

Breakdown of glucosylceramide by GBA2 generates ceramide: ceramide levels are normally very tightly regulated. Ceramide synthases (*CerS1-6*) are longevity-assurance genes in yeast (*Lag1-6*) assuring a balance between nutrient availability, autophagy and longevity, producing ceramide in the endoplasmic reticulum (ER) prior to transport to the Golgi or perinuclear ER, whereas GSL synthesis is carried out at the cytosolic surface of the Golgi. Ceramide levels in ALS are increased in the spinal cord and muscle in animal models and man [64,65,67,152], although decreased levels have been reported [66]. Furthermore, plasma levels of ceramides, particularly with fatty acid long chains (Cer22:1 and Cer 26:1), with linked reductions in sphingomyelins (Figure 1), are increased in ALS and can be used as biomarkers of progression [153]. Ceramide downregulates glucose transporters, particularly GLUT4 [154,155,156], producing mitochondrial dysfunction, and insulin resistance, which can be offset by pyruvate [157]. Indeed, ceramide has been proposed to ‘starve cells to death’ by nutrient transporter downregulation [157,158]. Thus, elevated levels of ceramide, via GBA2 activation or increased synthesis, may contribute to the dysfunction in glucose and lipid metabolism in ALS. Furthermore, ceramide levels are increased by ageing and increases have been strongly linked with sarcopenia [159]. Increased ceramide levels have been linked with senescence [160] and motor neuron senescence is a major factor in the ageing of primate spinal cord, with increased levels of senescence-associated β-galactosidase [161]. Inhibition of the rate-limiting enzyme for ceramide synthesis, serine palmitoyltransferase (SPT, Figure 1), inhibited ceramide formation, and a non-toxic inhibitor, ALT-007, reduced long chain GSLs and ceramides, with beneficial effects in mouse models of age-related sarcopenia [162].

A similar mechanism is causative for childhood ALS, in that variants of two subunits of SPT, SPTLC1 [163] and SPTLC2 [164] (the gate-keepers of GSL synthesis) favour changes in substrate from serine to alanine or glycine, increasing the formation of deoxysphingolipids (including 1-deoxyceramide), which are more resistant to degradation [163]. The variants of SPTLC1 linked to ORMDL isoforms are resistant to the negative feedback for sphingolipid synthesis caused by ceramide [163,165] (Figure 1), putting increased levels of ceramides (and 1-deoxyceramides) as potential causative factors in ALS. Consequently, the authors proposed specifically targeting the mutant SPTLC1 allele via siRNAs, and the use of SPT inhibitors, but the inhibitors myriocin, L-and D-cycloserine, imidazopyridine, and pyrazolopyridine are all associated with toxicity [166]. Serine supplementation would favour disease progression [164].

Ceramide (24:1) is a circulating biomarker for lower muscle mass in men, albeit not women [75]. Of the six ceramide synthases, ceramide synthase 1 (CERS1) is highly expressed in skeletal muscle (as is CerS5, which has been associated with ALS [49]), with CerS1 being specific for the synthesis of Cer18:1/18:0, the preferred substituents of GM1 [167]. CerS1 expression is progressively reduced with ageing [168]. Sphingosine, which is pro-apoptotic in high concentrations, was elevated four-fold in the spinal cord of transgenic Fus(1-359) mice at end stage, and associated with marked apoptosis; there was only a small increase in the antiapoptotic sphingosine-1-phosphate at end-stage [169]. Spinal cord sphingosine levels have also been linked with disease progression in SOD1^G86R^ mice [66]. Thus, dysregulation of ceramide/sphingosine may be highly deleterious in multiple pathogenic situations, as well as ALS (Figure 1).

Human iPSC motor neurons, astrocytes and microglia have very different ceramide profiles with differences in CerS1-6 expression and enzyme activity, resulting in different ceramide populations (chain length), with additional differences between cytoplasmic and nuclear populations [170]. In this study, *CerS1/4* (C18, C20) activity was ranked microglia>neurons>>astrocytes, *CerS2* (C22, C24, C26) microglia >> astrocytes > neurons, and *CerS5/6* (C14, C16) microglia>astrocytes>neurons. There were also differences between cortical, glutamatergic and GABAergic neurons [170]. Treatment with a GCS inhibitor increased ceramide levels, particularly in motor neurons [170], which may be a reason for the toxicity of this pathway in ALS models.

Ambroxol, by inhibiting GBA2, would inhibit the breakdown of GSLs, and prevent increased ceramide levels via this route, and protect against loss of GM1 and NMJs in SOD1^G86R^ [63] and TDP-43^Q331K^ [139] mice, whereas GCS inhibitors would increase ceramide levels [67]. The protective effects of ambroxol were equivalent to those of GM1 in motor neurons from SOD1^G93A^ mice, challenged with glutamate [139]. Ambroxol is also a powerful modulator of autophagy [171]. The drug is being studied clinically in ALS (NCT05959850). As ambroxol is also a chaperone for GBA1, thereby reducing a-synuclein aggregates, it is being explored clinically in Parkinson’s disease (NCT02941822, NCT05778617, NCT05830396, NCT06193421, NCT02914366, NCT05287503, NCT04388969), Lewy Body dementia (NCT04588285), and in neuronopathic Gaucher’s disease [172]. Parkinson’s disease evoked by GBA1 variants, may represent a distinct entity [173]. However, the transcripts of the protein-coding pseudogene of GBA1, GBAP1, which shares 96% homology in the coding region, are difficult to discern and appear to have non-lysosomal functions [174], which may undermine the otherwise well-defined mechanism for GBA1-variant Parkinson’ disease.

Thus, CMAH becoming a pseudogene, and consequent changes in the sialome, was a major event in human evolution, ~3–2.5 Mya, thereby changing the sialome, motor neurons, NMJs and cellular metabolism. This event merits further serious consideration in ALS, along with the role of GM1. Activation of GBA2 as a cause of denervation needs further confirmation. The proposition of Eisen et al. [175] that disruption of human-specific corticomotoneurones is one of the causes of ALS, may be compatible with these proposals, if the corticomotoneurones evolved because of CMAH becoming a pseudogene.

## 8. Key Role for Lipid and Lactate Metabolism in Human Evolution and ALS

Khaitovich’s group have shown that human lipid metabolism in cortex and skeletal muscle evolved to be distinct from that in chimpanzee and macaque monkeys [176], and the brain lipidome in humans is also distinct [60,177] from chimpanzee and macaque monkeys and mice. Changes in metabolism, perhaps to run, were a key event in human evolution [55,58]. The evolution of the metabolism, and musculature, between chimpanzees and humans is particularly striking [176]. Chimpanzees have more Type II (fast-twitch) glycolytic muscle fibres (66%) than man, being stronger, whereas hominins evolved to run, increasing endurance and the proportion of oxidative Type I (slow-twitch) muscle fibres (70%) over Type II (30%) [178]. However, as type II fibres predominantly metabolize and produce lactate, but are selectively lost in ALS [179], this may have brought about an increased risk of ALS, as lactate is a key controller of the ratio between glycolytic and lipid oxidative metabolism, and this is the basis of long-distance athletic performance [180,181]. Pro-BDNF is mainly produced in Type1 muscle fibres, yielding BDNF [182] and lactate potentiates BDNF signalling [183], implying an important lactate-BDNF cross-talk which would be changed in ALS. The brain’s metabolism is ~20% of total metabolic capacity in humans but <10% in chimpanzees, and chimpanzees and humans diverged in astrocyte to neuronal transport, with chimpanzees favouring lactate and humans glucose, as assessed by pathway enrichment analysis [184].

Weight loss is a critical parameter in ALS, due to muscle and fat loss, yet with increased energy expenditure [185]. What is happening?

Resting energy metabolism is higher in humans compared with other non-human primates [186], but is a further 14% higher in ALS patients at the very start of the disease, with increased oxidation of ketone bodies (β-hydroxybutyrate and acetocetate) and weight loss, compared to controls [187]. The role of skeletal muscle in ALS has been emphasized [179]. In this respect, SOD1^G86R^ mice, prior to the onset of denervation, show enhanced capacity for exercise with a reduction in glycolysis in fast twitch muscle with a switch to oxidative phosphorylation leading to hypermetabolism, resulting in an increase in glycogen synthase and glycogen content [188]. Similarly, in SOD1^G93A^ mice, hypermetabolism in skeletal muscle, switching to lipid oxidative metabolism, starts before disease symptoms [189]; furthermore, under these conditions there were increases in phosphorylated AMP-activated protein kinase pAMPK/AMPK and carnitine-palmitoyl transferase1 (CPT1), the latter driving lipid oxidation [189]. This results in an energy crisis as there were also declines in spinal cord mitochondrial complex 1 from 90 days, with more than 50% decline at 150 days. Home cage running wheels have greater precision than rotarod, and show that voluntary running declines at the very start of symptoms [190].

Changes in lipid metabolism are not restricted to mice with SOD1 mutations, because there are marked changes in tibialis anterior lipids in transgenic TDP-43^Q331K^ mice at a symptomatic age (P210), with reductions in acylcarnitine levels, presumably due to hyperactive oxidative metabolism via the rate-limiting enzyme for lipid oxidation, CPT1 [139]. Indeed, in end stage SOD1^G86R^ mice, triglycerides are almost completely depleted in plasma and muscle [65]. However, lactate is a major controller of the balance between glucose and lipid oxidation (see below).

## 9. Breakthroughs in Lactate Metabolism in Sports Science Which May Be Relevant to ALS

**Figure 4:**
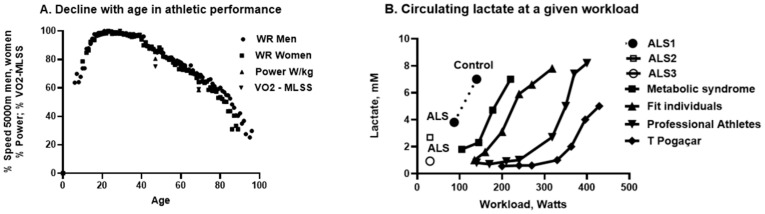
(**A**). Precise decline of world record (WR) human performance at 5000 m (where performance is close to VO_2max_) for men and women. Data are from [194] with permission and are calculated as the %age best WR. These data are from 2020, pre-COVID, as later data may be influenced by disparate availability of running shoes with improved efficiency. Note that when calculated as a %age of best performance men and women have superimposable declines. Data from a separate study in elite cyclists (average ages 25, 43 and65; tested for power, watts/kg, and VO_2_ at maximal steady-state lactate, MSSL) show an almost identical decline [195] emphasizing the tight coupling of performance, oxygen use and lactate metabolism with ageing. (**B**). Relationship between sustained power cycling, in watts, and venous blood lactate for patients with ALS (ALS1 [196], ALS2 [197], ALS3 [198]), metabolic syndrome, fit individuals, professional cyclists [180] and T Pogaçar [181]. The ALS1 patients were tested within three months of diagnosis. Data replotted from the references listed (with permission). As the ALS patients (and healthy controls) had an average age of 58, then an age-related decline of 15% (Figure 4A) would have contributed to their decline compared with the fit/professional cyclists (age ~30). Note the very low performance of ALS patients (~30 Watts) who had progressed further.

Evaluation of lactate metabolism is now a key factor in sports science, leading to recent remarkable advances in performance in endurance sports, with athletes and cyclists training specifically to increase mitochondrial efficiency at the lactate threshold for effects on oxidative phosphorylation [181], which is defective in ALS. San-Millan has proposed multiple zones of training intensity, with Zone 2 training, via prolonged low intensity exercise, used to stimulate mitochondrial content and function, and lactate metabolism. Exercise is the only physiological way to achieve this [191]. Zone 2 training has allowed absolute increases in human capacity, associated with better lactate clearance, as evidenced by cyclists such as the world champion, Tadej Pogaçar [181] (Figure 4). Metabolomics of endurance capacity in World Tour cyclists has shown that champion cyclists, such as Pogaçar (initially trained by San-Millan) have exceptional VO_2max_, lactate clearance and antioxidant capacity with associated metabolic profiles [192] (Figure 4). Pogaçar has demonstrated unmatched power over sustained periods (6.8 watts/kg, 448 watts for 40 min), being almost three minutes faster up a mountain (Plateau de Beille) in the Tour de France, than Armstrong (6.1 watts/kg) or Pantani, despite both of the latter using erythropoietin. This is an astonishing progression. What relevance does this have for ALS? While athletes and patients with ALS represent two extremes of the human condition (Figure 4B), can aspects of this spectrum be exploited therapeutically? Indeed, can lactate use be another measure of disease progression, or even exploited for therapy [193]? A decade ago, lactate dyscrasia was proposed as a driver for denervation and ALS [193], at a time when exercise was seen as a contributing factor. However, there are also arguments that the reverse is the case, and that lactate metabolism is disrupted, and should be supported, in ALS.

## 10. Lactate Shuttles and Energy Control

The old view of lactate being a metabolic waste product, limiting exercise capacity via the anerobic threshold, has been overturned, as mM levels are present in the blood, brain and muscle, with lactate being a major source of energy for the Krebs cycle in mitochondria after potential uptake by monocarboxylate transporter isoforms (MCT1-4). Lactate allows the uncoupling of mitochondrial energy generation via carbohydrates from glycolysis [199].

The ‘lactate shuttles’, described by George Brooks, circulate the energy substrate throughout the body and support gluconeogenesis [200,201,202]. The astrocyte-neuron shuttles were defined by Pellerin and Magistretti [203,204,205] and are critical to neuronal metabolism. After a meal, up to 80% of glucose may be converted to lactate in skeletal muscle and lactate is a major energy source for the heart, muscle, kidney and brain [206,207]. Furthermore, working skeletal muscle is only one source for lactate [208] which is also used for glycogenesis and to increase circulating glucose via the liver by the Cori cycle [209]. Glycolysis produces lactate (Ra) which is normally matched by oxidative metabolism (disposal, Rd) yielding blood lactate levels of 0.5–2 mM in normal resting conditions. However, endurance athletes can metabolize massive quantities of lactate as an oxidative fuel, while maintaining normal or only slightly higher blood levels (0.5–2 mM, Ra=Rd) up to 300 Watts, as training can double the mitochondrial mass, and thereby lactate disposal [181]. The maximum Rd recorded is by Tadej Pogaçar (blood lactate only 3 mM at 6 watts/kg for prolonged periods [181]; Figure 4B), who can maintain the necessary lipid oxidation while still maintaining gluconeogenesis, via lactate and the Cori cycle. The venous output of exercising muscle can be up to 13 mM lactate, while blood levels may be only 4 mM. Consequently, the term ‘anaerobic threshold’, due to lactate, is an outdated concept [210], and resting lactate levels are much less informative than are lactate stress tests. In contrast, power athletes using glycolytic fast-twitch muscles can generate >1000 Watts, even when aged (e.g., the 2024 English over 70s cyclocross champion, C Featherstone, personal communication), almost instantaneously from stocks of ATP/creatine phosphate, then glycolysis, but only for very short periods of time. This dual capacity of endurance and strength, depending on individual variation and niche specialization, would have given early hominins the capacity to adapt to multiple environments, perhaps outcompeting other hominins with intact CMAH. While little research has been performed on the links between CMAH loss and lipid oxidation, *Cmah*-null mice show upregulated gluconeogenesis, glycolysis, TCA cycle, and pentose phosphate pathways, but with resulting oxidative stress, because of their high metabolism [211].

The mitochondrial lactate oxidation complex is essential for the oxidation of lactate and pyruvate and comprises mMCT1, cytochrome oxidase (COx), basigin scaffolding protein (CD147), and the mitochondrial pyruvate carrier (mPC) and lactate dehydrogenases (LDH). Lactate is the preferred substrate over glucose in the heart either by metabolism to pyruvate or directly via mitochondrial transport by MCT1 (Slc16a1 [212]), with MCT1 being localized on the sarcolemma and mitochondria [201,213]. In the brain the relative roles of glucose and lactate have been extensively debated [204,214,215,216,217], but there is convincing evidence that lactate is the major energy substrate for rodent cortical neurons, increasing excitability and spiking via K_ATP_ channels in both excitatory and inhibitory neurons [218].

It was previously considered that lactate must generate pyruvate to enter Krebs cycle but Brooks has argued that lactate is metabolized directly by the mitochondrial reticulum as there are two lactate dehydrogenase pools (mitochondrial and cytoplasmic), and that the venous effluent of resting muscle has a lactate/pyruvate ratio at rest of 10–15 and of 20–25 during exercise, while in arterial blood the ratio was <10 but greatly elevated to 100–350 during hard exercise [219]. LDH-A favours pyruvate to lactate conversion, whereas LDH-B favours lactate to pyruvate conversion; inhibition of LDH-A increases TCA cycle activity [146]. The effects on the redox state of the cell can be major as pyruvate is reduced to lactate while NADH is oxidized to NAD^+^ (lactate + NAD^+^ ←→ pyruvate + NADH). The NAD^+^/NADH ratio has been proposed as a causative factor on the development clock speeds of different species, which is so evidently delayed in humans [146].

Lactate also inhibits circulating free fatty acid production via a G-protein coupled receptor, hydroxycarboxylic acid-1 receptor (HCA_1_, GPR81 [220], with an IC50 between 1 and 7 mM), which is highly expressed in adipocytes but also in the CNS, such as in interneurons and pyramidal cells of the hippocampus [221]. In hippocampal CA3 the effects of lactate on the excitability of CA3 pyramidal neurons are blocked by cholera toxin, although the effects of GM1 were not studied [222]: the authors hypothesized that the use-dependent, synapse-specific and transient rises in extracellular lactate they observed were important in strengthening synapses during pattern formation of memory. The interactions between the BDNF/TrkB and lactate/HCA_1_ signalling systems have been proposed to be critical for the synaptic transmission within the hippocampus and for its synaptic output [221].

Malonyl CoA is the major factor inhibiting lipid oxidation at CPT1, but high levels of lactate and pyruvate flood the mitochondrial reticulum during exercise, generating acetyl-CoA, lactyl-CoA and then malonyl CoA, thereby limiting lipid oxidation [192,223]. Lactyl-CoA can also ‘lactylate’ histone lysine residues and can lead to microglial activation, but also couple metabolism with gene regulatory networks [224]. In this respect, lactate is a major metabolic fuel for microglia, directly or via LDH catalyzing the reversible conversion of pyruvate and NADH to lactate and NAD^+^, with lactate being considered neuroprotective [225].

Astrocytes (Glut1; MCT1; MCT2; MCT4) have long been known to play a major role in neuronal metabolism via lactate [203] and this has been fully reviewed recently, the lactate coming from both cerebral and peripheral tissue [226,227]. Astrocytes are particularly enriched in MCT4. Glycogen derived from lactose has been shown to protect neuronal metabolism following exhaustive exercise [228]. Furthermore, glycogen is very rapidly broken down to produce lactate which is shuttled to neurons as fuel, within seconds of awakening or on demand. However, glycogenolysis is markedly reduced in spinal cord of SOD1^G93A^ mice and patients as symptoms progress [229]. Furthermore, there is a direct link with GM1, in that GM1 promoted glycolysis in astrocytes, promoting glucose uptake and lactate release; and enhancing neuronal mitochondrial activity, via lactate release [137]. Thus, in neuron-astrocyte cocultures, GM1 increased expression of seven genes involved in astrocytic metabolism including LDH-A, reducing pyruvate into lactate and the resulting NADH/NAD^+^ ratio [137]. However, it is not known whether astrocytic GM1 levels are impaired in ALS.

Oligodendrocytes and Schwann cells play a critical role in ALS as they are crucial for supporting motor neurons and myelination [230] and are particularly enriched in the lactate transporter MCT1, whereas neurons have MCT2 in the axons. While much attention has focussed on an accumulation of mutant SOD1, FUS or of TDP-43 cytoplasmic inclusions in oligodendrocytes, causing multiplication of precursor cells, and dysfunctional oligodendrocytes (reviewed in [231]), oligodendrocytes have immense energetic needs, necessary to synthesize myelin and support axons via lactate. Disruption of MCT1 in oligodendrocytes caused motor neuron toxicity, axonal damage and demyelination, in the motor cortex, but not other brain areas, of ALS patients showing a 50% decline in MCT1; MCT1 mRNA also declined in the spinal cord during the symptomatic stages in SOD1^G93A^ mice [232]. However, the reduction of oligodendrocyte MCT1 is critical for neurodegeneration and hypomyelination in aged animals but not early in life [233]. Thus, MCT1 is important for neuronal energy homeostasis in the ageing central nervous system (CNS), but MCT2 is found almost exclusively in neurons. The reduction in oligodendrocyte MCT1 that occurs with ageing may enhance the risk for axonal degeneration and atrophy in neurodegenerative diseases [233]. Disruption of MCT1 (but not MCT4) in Schwann cells leads to disruption of NMJs [234], implying a muscle/NMJ lactate cross-talk for NMJ maintenance.

The nodes of Ranvier are also critical hubs for metabolism, having ~5 fold more mitochondria than axons, and GM1 is particularly concentrated in lipid rafts at the nodes [235], which can be labelled with CTB (in competition with GM1 [236,237]). Antibodies to GM1 have been shown to accumulate in the nodes of Ranvier in ALS patients with lower motor neuron syndromes [238,239].

Schwann cells (Glut1; Glut3; MCT1; MCT4) myelinate peripheral motor neurons and maintain NMJs. Lactate is critical for function. Pyruvate kinase 2 diverts pyruvate to lactate dehydrogenase, and is upregulated in myelinating Schwann cells: deletion leads to neuropathy, impaired ATP production, and retraction of axon terminals, showing that lactate is essential for Schwann cells [240]. Futhermore, addition of dichloroacetate, to divert pyruvate to oxidative phosphorylation shows the critical role of aerobic glycolysis in Schwann cells [240]. The release of lactate to sciatic nerve axons (following glycogen breakdown) is essential for action potential generation [241]. Furthermore, lacate production by skeletal muscle must feedback to Schwann cells, where TDP-43 mislocalisation occurs in 70% of Schwann cells in ALS patients [242].

## 11. Lactate in Human Performance, Ageing and in ALS

A risk factor for ALS is age. The remarkable precision of the decline in oxidative performance with ageing in male and female athletes, shown in Figure 4A, represents the limits of homo sapiens (apart from very recent technical advances, such as carbon-plated running shoes) [243]. Figure 4A also allows the precise adjustment of performance with age. Why does the distance of 5000 m allow such a precise decline? [243]. First, age-related world records are performed by healthy individuals, so the variables of disease on ageing are minimized. Second, 5000 m run by athletes is sufficiently short to allow performance close (>90%) to VO_2max_ and maximal heart rate (>90%) while still running at maximal lactate steady-state (MLSS, Ra=Rd, 4–8 mM) [244]. Lactate oxidation is compromised in the elderly and this will contribute to the reduction in performance in such a precise manner [245]. This is the most precise decline in human performance with age published to date [243], which is further exemplified by a study in three groups of elite cyclists (average ages 25, 43 and 65 years old) which shows very similar declines in power (watts/kg) and VO_2_ at MLSS with age (Figure 4A) [246]: VO_2_max and MLSS were well correlated (r = 0.68). Thus, even highly motivated individuals, with extensive training, cannot escape a precise decline in performance, VO_2_, and MLSS with age, which has been proposed to follow a single exponential, following Moore’s law [247].

Figure 4B shows the profound differences in human performances and lactate levels between Pogaçar, professional athletes, fit people, subjects with metabolic syndrome, newly diagnosed ALS patients and longer-term patients. The control of lactate levels (Ra and Rd) are critical to performance and lactate is the main determinant of lipid versus glucose/lactate/pyruvate oxidation [180].

Reports of lactate stress tests in ALS are rare and only measure dysfunction at certain time-points and only one has explored newly-diagnosed patients to exhaustion, but this study is very informative [243,246] (Figure 4B). Unfortunately, it is not clear whether dysfunction is at the level of Ra or Rd, a critical question, although denervation of Type II muscle would impair Ra. Thus, mitochondrial dysfunction may increase lactate, wheres denervation would reduce it, meaning that sampling must be performed in well-defined conditions. Fortunately, the lactate threshold test (the work rate or VO_2_ where blood lactate starts to increase exponentially) is relatively easy to perform [247], although the low work rates in ALS are limiting. Skeletal muscle mitochondrial dysfunction is present from very early stages of the disease (within three months of diagnosis), is correlated to ALSFRS-R scores (except in patients with bulbar origin), and VO_2peak_ is reduced by ~40% [243,246]: peak power was reduced by ~38% and blood lactate was ~45% lower (3.8 +/− 1.9 mM versus controls: 7.0 +/− 2.9 mM), with a very strong, and linear relationship between power and lactate [243]. Heterogeneity between individuals was ascribed to motor neuron denervation differentially affecting oxidative/glycolytic muscle groups [243].

Thus, there is a major spectrum of the capacity to metabolize lactate between athletes (who train to double the mitochondrial mass of untrained people), diabetic patients and ALS patients (Figure 4B) and this spectrum seems under-utilized in measuring progression of the disease. With later stage patients, Finsterer showed that lactate stress testing was abnormal in 50% of ALS patients with average venous lactate levels of 1.42 mM prior to 15 min light cycling, 2.69 mM immediately after, and 2.18 mM 15 mins after [244]. Stress testing confirmed the abnormality was in skeletal muscle, relating to mitochondrial dysfunction [248], i.e., the reverse effects of the training of athletes. The lactate elimination rate was directly related to the speed of progression of ALS, and also directly to worse ALSFRS-R scores, indicating use in prognosis [245]. In this study, average lactate levels before 15 min of low-grade cycling (20−40 pedal rotations/min) were 0.62 mM (with a lactate to pyruvate ratio of 4.2), and immediately after exercise were 0.93 mM and 15 min after exercise, 0.76 mM. However, average basal venous lactate was not significantly different between early (0.75 mM) and late phases (0.78 mM) of ALS [245]. These values are very low (Figure 4B), and much lower than extracellular brain levels of 2 mM in rodents and 5 mM in awake humans, where lactate is present at 3-fold the level of glucose [249]. However, the lactate/pyruvate ratio is increased 3-fold in the CSF of ALS patients indicating mitochondrial dysfunction [69]. The circulating lactate/pyruvate ratio is also increased in ALS patients, associated with increased fatty acid levels and abnormal glucose tolerance [250].

A key question is therefore whether an initial increase in circulating lactate is due to poor mitochondrial metabolism, and a later decline due to progressive denervation? Is the decline in lactate production in ALS just a result of denervation, or a metabolic disorder which is central to the disease, particularly as lactate is an essential fuel, much of which is produced by skeletal muscle, which is increasingly denervated with disease progression? Lactate is a key driver of mitochondrial biogenesis, and regulator of fatty acid metabolism [251]. A key site for dysfunction are the membrane links between mitochondria-associated membranes (MAMS) and endoplasmic reticulum (ER-MAMS, Figure 3), which have been shown to be diminished in motor neurons with SOD1 mutations, where phenotype was partially restored with pyruvate [252]. Vance [253,254] discovered that the phosphatidyl inositol/phosphatidyl ethanolamine (PI/PE) ratio is critically dependent on the ER-MAMS (Figure 3), and critical for mitochondrial energy release. Phosphatidyl inositol serves as a basis for PE synthesis (and consequent phosphatidyl choline synthesis) and both neurodegenerative disorders and viral virulence are associated with changes in PI/PE metabolism [255]. The PI/PE ration has been reported to be changed in models of ALS [65,68].

Lactate levels of 1 mM cross the blood-brain barrier, supplying 8–10% of the brain’s energy requirements, whereas during vigorous exercise this can rise to ~10 mM and 20–27% of energy demand [202,256,257]. Thus initial denervation of Type II muscles may result in low lactate production, causing a feed-forward deficit, and affecting both carbohydrate and lipid metabolism.

There is now reasonable evidence that lactate metabolism may be critically involved in ALS, both in the periphery and CNS, where astrocytes and oligodendrocytes protect neurons with a lactate shuttle [209,225,227,258]. ‘Brain energy rescue’ has been proposed as a therapeutic concept to treat the neurodegenerative disorders associated with ageing [143,259]. In SOD1^G93A^ mice, disease progression is accompanied by lower blood lactate, perhaps showing the influence of low production, yet increased tibialis anterior muscle levels of lactate, and/or low capacity for utilization, lowered NAD^+^, NADH, and low MCT1 [260]; spinal cord lactate was decreased [261]. Reduced lactate release from astrocytes, together with activation of pro-NGF-p75 signalling in motor neuron death, was a major factor in mutant SOD1 toxicity, rescued by lactate (1–5 mM) [261]. Lactate, and pyruvate, also protect against glutamate-induced toxicity in cultured mouse cortical neurons [262]. Fibroblasts derived from both familial and sporadic patients have a different metabolic ageing profile, with defects in lactate metabolism, reversed by lactate [263].

The timing of increases in CNS lactate (and glycogen mobilization) are also tightly coupled to activity, doubling on awakening, reversing with sleep, linked to skeletal muscle activity [264]. Glutamate is a primary driver of astrocytic lactate release, linking ‘fuel’ supply with energetic requirements, and lactate offsets cortical excitoxicity caused by glutamate [265]. Noradrenaline, via α_1_-adrenoceptors, is also a major factor controlling lactate release and glycogen mobilization in the brain, linked to arousal, coupled to glutamate [198,200,266]. The electroencephalographic theta rhythm is disrupted in sensorimotor brain areas in the pre-motor phase of ALS patients, a potential measure of network impairment [267]. Deficits in theta-gamma phase amplitude coupling in SOD1^G86R^ and FusΔNLS/+ mice and ALS patients were associated with noradrenaline deficits in the brain [268]. Interestingly, low dose (but not high dose) antipsychotics such as clozapine, which are potent α_1_-adrenoceptor antagonists, are protective in ALS models [269], yet they increase theta rhythms in rats, from theta-generator cells in the stratum lacunosum molecular area of CA1 [270]. However, while lactate may be required in supporting the increased activity as a fuel, the complexity of its actions in these situations may be difficult to resolve in ALS.

Thus, in iPSC-derived motor neurons, lactate was reported to induce a pro-oxidative phenotype upon differentiation and increase pyruvate import—but the metabolic phenotype was unaffected by *FUS* mutations [271]. In contrast, iPSC-derived motor neurons (derived from *SOD1*^L144F^ and *TDP-43*^G298S^ patients) have been reported to have deficits in oxidative metabolism (caused by acetylation of complex I), driving glycolysis and increased lactate production [272]. In a post-mortem examination of ventral horn spinal motor neurons, mitochondrial electron transfer genes were down-regulated and in iPSC–derived motor neurons with *C9orf72* repeat expansions, mitochondrial transcripts were down-regulated, but boosting bioenergetics, via PGC1α, restored a normal phenotype showing metabolism to be a critical factor [273]. Thus while mitochondrial dysfunction has been shown to be an important factor in ALS [274], the rôle of lactate needs defining further. A major confounder is that the human brain evolved with intermittent starvation, and intermittent exercise [59], whereas chronically elevated lactate is associated with type 2 diabetes and cancer from the Warburg effect: chronically elevated lactate is deleterious to cardiac mitochondrial function [192]. Thus exercise, or timed L-lactate infusions, would be necessary for therapy.

Swim training slowed the loss of muscle strength [260], muscle mass [275] and increased blood lactate, NAD^+^, NADH, but without changing MCT1 or MCT4 levels in SOD1^G93A^ mice while the detailed mechanism is still unproven [276], swim training had beneficial effects on glycolytic activity in tibialis anterior muscle.

There may be links with the proposition of Eisen et al [175] that the evolution of corticomotor neurons leads to a susceptibility of ALS, as intensive endurance exercise, (peak lactate 10–12 mM in athletes) or lactate infusions, leads to increases in motor cortex excitability (resting motor threshold) in order to delay the onset of fatigue [277,278], presumably due to an interaction with K^+^_ATP_ channels [222]. However, cortical hyperexcitability precedes the development of symptoms in some carriers of SOD1 mutations and early in sporadic patients [175,279,280], and cortical hyperexcitabiliity has been associated with shorter survival times [279]. However, hypoexcitability has been observed to progress with time, and finally to cortical inexcitability [279], presumably at a time when peripheral lactate production is also low, because of denervation. Repetitive transcranial magnetic stimulation of the motor cortex will also increase blood lactate, some of which is of central origin [281,282]. Some attentional and working memory tasks were reduced at the time of the changes in motor neuron excitability leading to the proposition that, in the CNS, lactate can also perform as a neuromodulator [278]. It is thus important to define whether lactate is beneficial or deleterious for ALS patients, in the CNS and periphery, and precisely how this is linked with lipid/glucose metabolism.

## 12. Previous Exercise as a Risk for ALS: Subjects with C9orf72 Expansions May Be Outliers?

ALS is one of the rare human diseases where physical activity has been claimed to be a risk factor, and in a comprehensive review of 93 studies, strenuous physical activity was proposed to be a risk factor for ALS [283]. Long-term physical activity (an extra 10 kJ/kg/day) was reported to increase risk, with an odds ratio of 1.47 [283]. However, in a prospective cohort study, as opposed to case control studies, physical activity was weakly inversely associated with ALS mortality [284] and recent studies have also supported this [285].

Much of this controversy may have been resolved as some ALS risk genes such as *C9orf72* causing G4C2 repeat expansion, are involved in the response to exercise, and exercise with this genotype shortens the age of onset, so exercise-induced ALS is associated with a risk-genotype [286], with the possibility that a certain dose of exercise may induce ALS in subjects with *C9orf72* expansions. Presymptomatic patients with *C9orf72* mutations have a considerably lower body mass index, which coupled with poor nutrition and skeletal muscle loss results in NfL elevation [287]. These are important findings, amplified by a study in peripheral blood mononuclear cells (PBMCs) in response to exercise where 72 pathways were associated with rare ALS variants: NGF and FGF signalling pathways were the most significantly involved, whereas changes in BDNF signaling were not included in the changes. However, BDNF signalling is impaired in fast motor neurons in SOD1^G93A^ mice, reducing axonal transport, with upregulation of truncated TrkB and p75^NTR^ receptors [288]. These effects may be specific to BDNF, as boosting the BDNF levels (but not of other neurotrophins) in muscle increased the axonal transport in Charcot-Marie-Tooth mice (CMT2D [289]). Axonal vesicles rely on glycolytic metabolism, because while LDH-B is the main neuronal isoform, LDH-A is expressed on vesicle surfaces, so axonal transport depends on lactate, and the NADH to NAD^+^ ratio [290]. GM1 is lost from NMJs at the start of denervation in SOD1^G86R^ mice [66,73], when GBA2 is increased [63], potentially disrupting both lactate metabolism and neurotrophin signalling via TrkB [133,140,291,292,293] and TrkA receptors [126]. While it is not yet proven that the GM1/BDNF link is a critical pathway in ALS, it is possible that the TrkB-induced improvement in RCI/mitochondrial efficiency (not shared with NGF/TrkA signalling) may be involved in the protective effects. In this respect, analysis of mitochondrial function, via haplotypes, indicated that function modified survival but not risk [294]. However, the effects on BDNF on mitochondrial function are blocked by IL1β and so may be more susceptible to the more inflammatory signatures occurring in genetically driven cohorts, such as *C9orf72* expansion [295].

Furthermore, if genetic variants such as *C9orf72* are directly contributing to the exercise ‘dose’ which can be tolerated prior to incidence, then previous studies, not measuring variants, will have overestimated the effects of exercise on ALS incidence in sporadic patients, undermining protective effects. Up to 40% of fALS and 6% of sporadic ALS may be due to *C9orf72* expansions so the effects of exercise in sporadic patients need to be re-evaluated. If the exercise ‘dose’ is important then a portion may be due to stress. Can the sport stressor also influence incidence?

Different sports create different stressors: the shock of impact in football both in American (standard mortality rates, SMR 4.3 [296], SMR 3.9 [297]) and European (SMR 6.5 [298]) football has led to differential potential risks of ALS [299,300,301]. Stressors may also explain the finding that only the fastest long-distance cross-country skiers in the Swedish Vasaloppet cohort had a 4-fold risk of ALS [302], as these athletes would train for long periods in the cold, using glucose supplements as an energy source, when high carbohydrate and low fat intake are associated with risks of ALS [303]. Even running has a high degree of impact. The vertical reaction force per foot strike is 2–2.9 times body weight when running [304], so the author, who has run a lifetime 126,000 kms with an average stride length of ~1.6 m and a weight of 64 kg has cumulated >10 million tons of foot shock, whereas these forces are buffered when cycling, which has a low association with ALS [298].

Deconditioning following bed-rest, reduced exercise following lock-down, or reduced exercise capacity, will rapidly down-regulate mitochondrial function, and is a major factor for injured athletes, and the elderly [305]. Three to five weeks of muscle immobilization causes a loss of nearly half of normal strength [306] and the same factors apply to denervated muscle in ALS patients, where deconditioning has been proposed as the main cause of impaired responses to exercise [246]. Such deconditioning in man is accompanied by accumulating intramuscular triglycerides (the opposite to effects seen in SOD1^G86R^ mice [65]) and lipotoxic ceramides [307] (as seen in SOD1^G86R^ mice [65]). Elimination of serum lactate in ALS patients is compromised in bicycle exercise lactate stress tests [244,248,308] and the degree is related to disease progression [245]. Thus, there is a major spectrum of the capacity to metabolize lactate between athletes (who may have double the mitochondrial mass of untrained people), diabetic patients and ALS patients (Figure 4B) and this spectrum seems under-utilized in measuring progression of the disease. Furthermore, lactate promotes PGC1α (and BDNF and transcription factor EB- TFEB) in the brain, which regulates mitochondrial biogenesis, but the effects of lactate were mitigated by *FUS* mutations [309]. There is thus evidence that lactate metabolism is pathologically reduced in ALS, but more patient studies are needed.

Park et al [310] reviewed whether therapeutic exercise can slow down decline in ALS patients but the exercise was quite intensive, and while some effects were noted on respiratory function, little effects were noted on limb function, perhaps because exercise is effective predominantly in the early stages of denervation [311]. Thus while Bello-Haas et al. [312] found beneficial effects in a small trial of resistance exercise in ALS, in a Cochrane review in 2013, they concluded that more research was needed to conclude [313]. In another small trial light exercise tailored to the individual was beneficial [314,315], while in SOD1^G93A^ mice intense aerobic exercise was deleterious [316], dismantling NMJs in tibialis anterior. However, there is a possibility that the low lactate efflux from denervated or partially denervated type II fibres may be a cause of spreading denervation to other muscle groups, i.e. facilitating progression. There exists a specific lactate/BDNF synergy between type II (lactate) and type I muscles where pro-BDNF is present. Lactate increases mature BDNF release [182]: both are important for NMJs. Furthermore, lactate promotes mitochondrial biogenesis regulating fatty acid metabolism [251], and BDNF increases mitochondrial respiratory coupling index [142,143,317]. Both lactate and BDNF are required for axonal transport in motor neurons [288,318]. In this respect, loss of NMJs with associated functional decline is a hallmark of advanced human ageing over 75 years old, associated with mitochondrial dysfunction [319]. However, octogenerian master athletes, with regular exercise, have more motor unit numbers and better NMJ stability in tibialis anterior muscle [320]; female octogenarian athletes had superior reinnervation capacity whereas frail/prefrail women had neurogenic muscular atrophy, putting NMJ stability as a critical factor in functional ageing [321]. Functional connectivity in master athletes was maintained in both motor and cognitive areas [322]. Nearly all serious athletes use regular interval training to boost performance and lactate levels remain high throughout interval training sessions in athletes despite great fluctuations in heart rate [323]. These interval sessions would increase circulating levels of both BDNF and lactate [324] and peripheral lactate induced by exercise has been shown to increase hippocampal BDNF expression (via sirtuin-1 deacetylase) [325]. Lactate may be the reason for this NMJ integrity, despite the decline in MLSS, as conditional deletion of MCT1 (but not of MCT4) in Schwann cells caused disrupted NMJ innervation in mice, partially offset by upregulation of genes involved in oxidate metabolism [229]. Thus, evidence from sports science indicate that modulating lactate production is a major factor for assuring performance in athletes, but while the situation is still unclear in ALS, there are many indirect indications of potential benefit which should be exploited therapeutically.

## 13. Future Directions for Research and Therapies

1. The critical path for glycosphingolipid and ganglioside pathways is under-researched, especially as these pathways provide useful biomarkers. Much of the research of the effects of GM1 have involved TrkA, yet effects on TrkB in lipid rafts may also be important. Furthermore, which the effects of Neu5Ac-GM1 and its oligosaccharide have been well studied, direct comparisons with Neu5Gc-GM1 are lacking.
  While GM1 is a critical component of the inner nuclear membrane, associated with the Na^+^/Ca^2+^ exchanger [136], there are no studies on whether it has an impact on TDP-43 accumulation in the cytoplasm.
2. The pivotal role of GCS, GBA1 and GBA2 has indicated ways forward which are under scrutiny. While inhibitors of the super-enhancer gene UGCG (GCS) have proven deleterious in ALS models [65,67], this has increased focus on GBA2 inhibitors, several of which are in development. The use of CTB, to evaluate GM1 and related structures, will be important in the future, as will the use of CTB-S to cause very specific denervation of motor neurons. The effects of GBA2 inhibitors as glucosyltransferase inhibitors, thereby glycosylating cholesterol in lipid rafts, may impact lipid raft stability with major implications. The status of the GBA2 inhibitor/GBA1 chaperone, ambroxol, has been reviewed above. However, most GBA2 inhibitors are in development for Parkinson’s disease: ALU1811 is in preclinical development by Biogen and Alectos for Parkinson’s disease, and the GCS and GBA2 inhibitor nizubaglustat is in development for PD [326]. While miglustat is predominantly an inhibitor of GCS, its inhibition of GBA2 has been speculated to be responsible for lack of deleterious effects [327]. Endogenous surfactants, such as saposin-C, may cause profound changes in GBA1 and GBA2 activity and drugs such as ambroxol increase saposin-C levels [328]. The very recent discovery that GCS is a super-enhancer [70] means that much research will be necessary to redefine the effects of GCS inhibitors.
3. The breakthroughs in knowledge about the critical role of lactate in human performance should be applied to ALS research. The studies reviewed above do not provide definitive answers as to whether Ra or Rd are specifically affected in ALS and whether peripheral lactate may be neuroprotective. Sports scientists have perfected dried blood-spot collection, which can be easily transported, to assess lactate, carboxylic acids, fatty acids and acyl carnitines regularly in racing cyclists [181], and this relatively simple technology could be used to assess the metabolic position and progression of ALS patients. Specific low-impact training, tailored to each patient, to down-regulate lipid metabolism, and increase glucose metabolism and mitochondrial function has been recently reviewed and proposed [315], as is the case with athletes (Figure 4). However, as lactate can be increased by mitochondrial dysfunction, but reduced by denervation, conclusions can only be reached with protocols defining MLSS or lactate at exhaustion. So, can a programme like phase 2 training (low intensity training in cycling), to increase mitochondrial efficiency and lactate metabolism (Figure 4), restore glycolytic activity, and have an effect in ALS on lactate use and energy restitution, with perhaps protection against further spreading denervation? Patients with C9orf72 mutations (and probably patients with other highly penetrative mutations for which exercise is a ‘stressor’) may not benefit in comparison with sporadic patients. Intensive cycling has a low risk of ALS [298], despite requiring more hours of exercise than other high-intensity sports. Light cycling may produce the metabolic resilience and improved lactate metabolism associated with stage 2 training. But how can exercise be performed at the beginning of denervation? There are now available motorized exercise bikes, such as Motomed (https://www.motomed.com/en/, accessed on 1 October 2025.) allowing muscles to be stretched, doing minimal work without applying force, with spasm movement protectors. Lactate is increased even with passive cycling [329] and it seems important to perform trials of prolonged passive cycling (or powered arm exercises)/day with measures of lactate use [330,331]. This may allow the muscles to maintain measures of mitochondrial efficiency and produce lactate, even when denervated or denervating. Recently, dynamic cycling sessions have been shown to ameliorate subthalamic function in patients with Parkinson’s disease [332]. Spectacular results have been found using physiotherapy in dogs with degenerative myopathy (diagnosis at ~9 years and a mean survival time of 55 days) [333]: moderate physiotherapy increased survival to 130 days, and intensive physiotherapy to 255 days. Remote ischaemic conditioning has been used to increase lactate in a variety of disorders, by inflating blood pressure cuffs to 20 mm Hg above systolic pressure for four 5-min occlusions in affected limbs.
4. Viral infections prior to the development of ALS. While viral infections have not been associated with ALS, the entry and exit of enveloped viruses are dependent on the same GSLs and GM1, and the associated enzymes. GCS, as a super-enhancer, is up-regulated following viral infection [70]. Viral infections also remodel lipid metabolism in similar ways to ALS, but via remodelling endoplasmic reticulum for replication and creation of viral envelopes [334]. It is only recently that multiple sclerosis has been linked to prior Epstein-Barr virus infection, despite an odds-ratio of ~30 [335]. As ALS may develop late after injury, prior viral infection would be missed.
5. Combination with therapeutic agents may also be beneficial in order to promote reinnervation or protect from denervation. Therapeutic approaches targeting skeletal muscle, with efficacy in preclinical models, have been recently reviewed [336]. Drugs changing GSL metabolism are an obvious target [337]. However, some agents which had been developed for changing cardiac metabolism are being examined for ALS in the clinic. Thirty five years ago, switching cardiac lipid oxidation to glucose fuels was a major drug discovery topic, as ischaemia prevented full oxidation of lipids causing build-up of acylcarnitines and lysophospholipids [338,339,340]. Ranolazine is cardioprotective by effects on pyruvate dehydrogenase and reducing excess lipid oxidation [341,342]. The drug is being developed for cramps in ALS (NCT06527222). Trimetazidine [90,343] is a forty-year old antianginal drug, which modulates mitochondrial metabolism and inhibits long-chain mitochondrial 3-ketoacyl coenzyme A thiolase, a key enzyme in lipid oxidation [344,345]: the drug has been banned from use in sports because of extensive doping allegations linked to its effects on cardiac energy metabolism [346]. Trimetazidine extended survival of SOD1^G93A^ mice and protected NMJs reducing motor neuron loss [343]. The drug is being studied for effects on metabolic flexibility in ALS (NCT04788745), although it is contra-indicated in Parkinson’s disease [347]. These metabolic approaches may be particularly effective coupled to personalized exercise programs. However, as these drugs are being repurposed, development is not easy [348] and a coordinated approach by the ALS community and sponsors may be necessary for registration if clinical trials are positive.

## 14. Conclusions

ALS may be a consequence of human evolution following CMAH inactivation (probably by a pathogenic infection), enabling endurance hunting, running, expanding the central nervous system, and engendering the changes in lipid and glucose/lactate metabolism required to support these activities, which may open up new therapeutic opportunities for ALS. However, some highly penetrative mutations (e.g. *C9orf72* expansions) may still be resistant to this approach.

## Figures and Tables

**Table 1:** Evolutionary changes from Australopithecus, 3.2–2.5 Mya, through to homo sapiens.

CMAH becomes a pseudogene, the sialic acid Neu5Gc replaced by Neu5Ac, driving neurotrophins [53,57,58], changing NMJs, metabolism, facilitating running [15,55], * Siglecs (receptors for sialic acids) adapt to Neu5Ac [32], * Major changes in lipid metabolism to increase VO_2max_, and endurance [59], * Doubling of copy number of Olduvai protein domains from *NBPF* genes (1q21.1, paired with *NOTCH2L*) in the last 1–3 Mya to reduce neuronal metabolism in development, extending the window of neurogenesis (linked to IQ, schizophrenia, autism) [60]. The protein from the human-specific gene *ARHGAP11B*, when transported into the mitochondrial matrix; stimulates glutaminolysis, in conjunction with GLD2, increasing neocortex size [61]. Delayed brain development and adolescence, and hence greater potential longevity, * Human brain uses ~20% of metabolic resources, whereas <10% in chimpanzees, due partly to differential gene expression in astrocytes for glucose and lactate transport [62], * Voice box (pharynx) develops, allowing speech, with specialization of bulbar motor neurons [54], * A 50% reduction in forearm mass, with increased shoulder width, compared with chimpanzees, allows the arm movement associated with running, associated with changes in lipid metabolism and less Type II muscle [54] *, Fifty % longer legs relative to body weight, from austrolopithecus to homo habilis, with smaller toes [54], Increased size of spinal and gluteal muscles, which are crucial for upright running [54], Long tendons are connected to short muscles, and the human Achilles tendon and sprung plantar arch save ~50% of the metabolic cost of running, mainly optimized in homo habilis, (note: the short Achilles tendons and body size of Neanderthals indicate that they did not run [54]). Heat-tolerance with loss of body hair and capacity to sweat at up to 3.5 litres/hour, the greatest heat dissipative capacity of any animal. Animals that lose heat by panting cannot pant and gallop, allowing hunting by heat stress [52,53], Increased cranium size, increased tracking and cooperative ability, and ability to plan effort for endurance, rather than living in the ‘eternal present’ which limits the endurance capacity of animals [54].

* of potential relevance to ALS.

**Table 2:** Potential effects of GM1 on human evolution and neuronal development, of relevance to ALS.

Present in CNS of all mammalian species, representing about 17% of all gangliosides, CMAH becomes a pseudogene 3.2–2.5 Mya, Neu5Gc replaced by Neu5Ac in gangliosides, yielding human-specific GM1 and related gangliosides [34], probably caused by a pathogen. Key component in lipid rafts, critical for cell signalling [130], but targeted by viruses. Antibodies to GM1 and related gangliosides found in motor neuropathy, and in Guillain-Barré patients (and associated with poor outcome [132]), Binding site for GM1 on TrkA receptors described with two sites, a transmembrane one for ceramide and an extracellular one for the oligosaccharide [124], Absence of GM1 in NG-CR72 cells prevents TrkA membrane expression and responses to NGF, restored by GM1 [125], Activation of TrkB receptors directly with effects equivalent to BDNF, yielding neuroprotection [133], Cholera toxin bound to saporin (CTB-S) causes specific motor neuron death by retrograde suicide transport to motor nerves expressing GM1 [89]. GM1 found in nuclear envelopes with a Na^+^/Ca^++^ exchanger, modifying gene expression [134,135,136], Complex effects on α-synuclein aggregation [114,115], Profound effects on astrocytic/neuronal lactate metabolism [137], and strategically located in nodes of Ranvier, Increased mitochondrial density, cristae and oxygen consumption in Neuro2A cells [138], GM1 is lost from NMJs at the beginning of denervation [73,139].

## Data Availability

No new data were created or analyzed in this review. Data sharing is not applicable to this article.

## Abbreviations

LDH	lactate dehydrogenase
LacCer	lactosylceramide
ERK	extracellular signal-regulated kinase
CPT1	carnitine-palmitoyl transferase1
CMAH	cytidine monophospho-N-acetylneuraminic acid hydroxylase
CTB-S	cholera toxin β-subunit bound to saporin
COPT	chronic obstructive pulmonary disease
CTB	cholera toxin β-subunit
BDNF	brain-derived neurotrophic factor
Bcl-2	B-cell lymphoma-2
ALSFRS-R	Amyotrophic lateral sclerosis functiona rating scale-revised
ALS	Amyotrophic lateral sclerosis
iPSC	induced pluripotent stem cells
HILIC–ESI–MS/MS	hydrophilic interaction, electrospray ionization tandem mass spectrometry
HCA_1_	hydroxycarboxylic acid-1 receptor
GSL	glycosphingolipid
GM1	monosialotetrahexosylganglioside
GlcNAc	N-acetylglucosamine
GCS (or UGCG)	Ceramide glucosyltransferase
GBD	Ganglioside-binding domain
GBA2	non-lysosomal β-glucosylceramidase
GBA1	lysosomal β-glucosylceramidase
GalNAc	N-acetylgalactosamine
FUS	fused in sarcoma RNA-binding protein
GlcCer	glucosyl ceramide
GalCer	galactosyl ceramide
FGF	fibroblast growth factor
FAPP2	phosphatidylinositol-four-phosphate adapter protein 2
MAPK	mitogen-activated protein kinases
MLSS	maximal lactate steady-state
NGF	nerve growth factor
NMJ	neuromuscular junction
MAPK	mitogen-activated protein kinase
MCT	monocarboxylate transporter isoforms
MEP	motor evoked potential
MND	motor neuron disease
Mya	million years ago
Neu5Ac	N-acetylneuraminic acid
NfL	neurofilament light chain
NGF	nerve growth factor, NSC: neural stem cell
ORMDL	sphingolipid biosynthesis regulator
pAMPK	phosphorylated AMP-activated protein kinase
PGC1a	peroxisome proliferator-activated receptor gamma coactivator 1-alpha
PPAR	peroxisome proliferator-activated receptor coactivator
Ra	lactate production rate
Rd	lactate metabolism rate
RCI	mitochondrial respiratory coupling index
SICI	short interval intracortical inhibition
Siglecs	sialic acid-binding immunoglobulin-type lectins
SOD1	superoxide dismutase 1
SPT	serine palmitoyl transferase
SSEA	stage-specific embryonic antigen
TDP-43	transactive response DNA binding protein of 43 kDa
TrkA	tropomyosin receptor kinase A
TrkB	tropomyosin receptor kinase B
UGT8	ceramide galactosyltransferase
VDAC1	voltage-dependent anion channel 1
